# *XRCC4 c.1394G>T* Single Nucleotide Polymorphisms and Breast Cancer Risk among Filipinos

**DOI:** 10.31557/APJCP.2019.20.4.1097

**Published:** 2019

**Authors:** Julius Adrie Garcia, Noel Angelo Kalacas, Teresa Sy Ortin, Maria Cristina Ramos, Pia Marie Albano

**Affiliations:** 1 *The Graduate School,*; 2 *Research Center for the Natural and Applied Sciences, *; 4 *College of Science, University of Santo Tomas, *; 3 *University of Santo Tomas Hospital, Benavides Cancer Institute, Manila, Philippines. *

**Keywords:** XRCC4 SNP, breast cancer, PCR-RFLP, Filipinos

## Abstract

**Background:**

The identification of cancer-associated single nucleotide polymorphisms (SNP) and mutation genes is a promising approach in recognizing individuals who are at risk of developing cancer. Hence, this study was conducted to determine the association between *XRCC4 c.1394G>T* SNP and breast cancer development among Filipinos.

**Methods:**

Genotyping for *XRCC4 c.1394G>T* SNP was performed on breast cancer patients (n=103) and their age- and sex- matched clinically healthy controls (n=103) by polymerase chain reaction – restriction fragment length polymorphism.

**Results:**

Significant difference in genotype (p=0.007) and allele (p=0.003) frequencies in *XRCC4 c.1394G>T* was observed between the breast cancer cases and controls. Carriers of the *XRCC4 c.1394 G>T* genotype were observed to have significantly higher risk of developing breast cancer compared to individuals with T/T genotype (OR=2.67, 95% CI: 1.36 – 5.25). *XRCC4 c.1394G>T* combined with passive smoking may also significantly increase risk of breast cancer (OR=14.73; 95% CI= 9.88-18.86).

**Conclusion:**

*XRCC4 c. 1394G>T* may be associated with breast cancer development among Filipinos.

## Introduction

Breast cancer remains the leading cause of cancer among women worldwide (Sassi et al., 2013). Epidemiological studies show that breast cancer is a multifactorial disease associated with exposure to ionizing radiation, tobacco smoking, alcohol consumption, and use of hormones or oral contraceptives (Dumitrestcu et al., 2005). Furthermore, it is widely established that breast cancer could result from a series of genetic alterations that disrupt the normal mechanism which controls cell proliferation, differentiation, apoptosis, and genomic stability (Wu et al., 2008). To counterbalance the detrimental effects of DNA damage, cells have established a dedicated DNA repair system, which can be classified into few distinct mechanisms based on the degree of lesion in the DNA (Bau et al., 2011).

The most biologically perilous types of DNA damage are called the double strand breaks (DSBs). A single unrepaired double strand break is reasonable to induce cell death. These events have been linked with the progress of many types of cancer or other genomic defects (Dexheimer, 2013). The two important mechanisms in DSBs are (1) homologous recombination (HR) and (2) non-homologous end-joining (NHEJ). The two repair systems require a different necessity for a homologous template DNA and also the fidelity of DSB repair. HR-directed repair is an error-free mechanism because it utilizes the genetic information confined in the undamaged sister chromatid as a template (Li and Heyer, 2008). On the other hand, NHEJ is error-prone and involves elimination of DSBs by direct ligation of the broken ends. In all phases of the cell cycle, NHEJ is the principal pathway in mammalian cells while HR is constrained to the late S and G2 phases (Lieber, 2010).

Several studies have shown that some genetic variants of DNA repair genes, such as the X-ray cross-complementing group 4 (*XRCC4*) gene are associated with breast cancer pathogenesis (Chiu et al., 2008). The *XRCC4* gene which encodes for XRCC4 protein is involved in non-homologous end joining (NHEJ) mechanism critical in double strand break repair (West et al., 2000). The XRCC4 protein helps in repairing the DNA double-strand breaks by stimulating the ligation of non-complementary and complementary DNA ends using XRCC4 ligases (Ming- Zhonget al., 2015). Furthermore, this protein plays a pivotal role in the completion of VDJ recombination in order to generate antigen receptors that can collectively recognize different types of molecules (De Fazio et al., 2002).

Meta-analyses have shown the association of polymorphisms in *XRCC4* gene specifically the G>T variant with increase in breast cancer risk among Asians and Caucasian, notably in Chinese, Korean, and American populations (Fu et al., 2003; Lee et al., 2005; Han et al., 2009). Furthermore, numerous studies have reported that SNP in the *XRCC4* gene is associated with risk of developing breast (Bau et al., 2004), skin (Han et al., 2004), gastric (Chiu et al., 2008), liver (Mederacke et al., 2013), and oral cancer (Yen et al., 2008). However, it must be noted that their findings were also race-specific. Hence, this study is the first to present the possible association of *XRCC4 (c.1394G>T)* polymorphism with the risk of breast cancer among Filipinos.

## Materials and Methods

*Study Population*

Filipino patients, 18 years old and above, with histologically confirmed breast cancer, either newly diagnosed, receiving treatment, or in remission, seen at the University of Santo Tomas Hospital (USTH), Manila between December 2015 to April 2016, were recruited for the study. The cancer patients (cases) were age- (±2 years) and sex-matched with volunteer controls who were assessed by a physician collaborator to be clinically healthy and not suspected to have any type of malignancy. Both cases and controls were asked to accomplish through interview a standardized questionnaire inquiring on their risk factors (diet, alcohol and tobacco use, medical history, family history of cancer, reproductive health, environmental and psychological factors, and sedentary behaviour). Five (5) mL peripheral blood collected from the participants were immediately stored at -80°C until molecular analysis. Clinical data of the cases (age at cancer diagnosis, tumor site, tumor grade, TNM stage, and treatment received) were retrieved from clinical records and histopathology reports. 

Ethical clearance was obtained from the Institutional Review Board (Protocol Reference No. IRB-MD-09-2015-133) of USTH and all participants gave their written informed consent. 

*Genotyping*

Genomic DNA was isolated from the blood samples of all study participants using ReliaPrep™ Blood gDNA Miniprep System (Promega, USA) following the manufacturer’s protocol. All samples were genotyped in triplicate with consistent results to confirm that the genotypes have no mixture of germline and somatic mutations. The genotype at the polymorphic locus of *XRCC4 c.1394G>T* was analyzed by polymerase chain reaction – restriction fragment length polymorphism (PCR-RFLP), and further processed as described (Chiu et al., 2008a, Ming-Zhong et al., 2015) but with minor modifications with the annealing temperature and duration of digestion.

A single PCR reaction comprised of a 20 μl total volume of 10 μl of PCR master mix (20 mM Tris-HCl, pH 8.3, 50 mM KCl, 1.5 mM MgCl2, 0.2 mM of each dNTPs, 2.5 mM U Taq DNA polymerase), 0.5 μl each of 0.25 μM forward and reverse primers, 7 μl nuclease free water, and 2 μl DNA (89 ng/μl). Target gene was amplified using the primers 5’-GATGCGAACTCAAGATACTGA-3’ (forward) and 5’-TGTAAAGCCAGTACTCAAATT-3’ (reverse) under the following conditions: initial cycle at 94°C for 5 min; 40 cycles of 94°C for 30 s, 52.8°C for 30 s, and 72°C for 30 s; and a final extension at 72°C for 10 min. PCR products were digested overnight with *HincII *at 37°C. The digested amplicons were subjected to 12% polyacrylamide gel electrophoresis for 60 min at 100V and viewed under a UV transilluminator. Expected fragment sizes were as follows: T/T: 300 bp; G/G: 200, 100; G/T: 300, 200, and 100 (Ming-Zhong et al., 2015).

*Statistical Analysis*

To ensure that the controls used were representative of the general population and to exclude the possibility of genotyping error, the deviation of the genotype frequency of *XRCC4 SNP* in the control subjects from those expected under the Hardy-Weinberg equilibrium was assessed using the goodness-of-fit test. Pearson’s Chi-square test or Fisher’s exact test (when the expected number in any cell was less than five) were used to compare the distribution of the *XRCC4* genotypes between cases and controls. Epidemiologic risk factors associated with the genotypes were estimated as odds ratio (ORs) and 95% confidence intervals (CIs) using unconditional logistic regression. Data was recognized as significant when the statistical p-value was less than 0.05. All statistical tests were performed using SAS, Version 9.1.3 (SAS Institute Inc., Cary, NC, USA) on two-sided probabilities.

## Results

A total of 206 samples (103 histologically confirmed breast cancer cases matched with 103 clinically healthy controls) were included in this study. Majority of the cases were 41 to 50 years old, had either well-differentiated (G1) or moderately-differentiated (G2) tumor, and were in their early stage of cancer (Tis, T1, or T2) at the time of diagnosis (Table 1).

**Table 1:** Demographic and Clinical Characteristics of Breast Cancer Patients

Characteristic	n=103	%
Sex		
Male		
Female		
Age at initial diagnosis		
< 50	47	45.63
≥ 50	56	54.37
Tumor grade		
Grade cannot be assessed	12	11.65
Well-differentiated	34	33.01
Moderately-differentiated	35	33.98
No data available	22	21.36
Tumor stage		
Primary tumor cannot be evaluated	8	7.77
Tis, T1, and T2	52	50.49
T3 and T4	33	32.04
No data available	10	9.71
Treatment received		
None	10	9.71
Immunotherapy	15	14.56
Surgery	4	3.88
Radiation therapy	12	11.65
Chemotherapy	21	20.39
Surgery and chemotherapy	8	7.77
Surgery and radiation therapy	9	8.73
Chemotherapy and radiation therapy	4	3.88
Surgery, chemotherapy, radiation therapy	17	16.51
No data available	3	2.91

**Figure 1:**
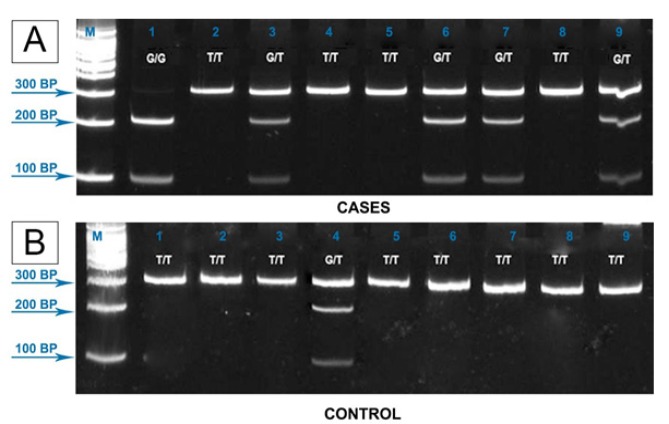
Representative Results for *XRCC4 c.1394G>T* genotyping by PCR-RFLP Method. DNA isolates from breast cancer cases and controls were subjected to PCR-RFLP using *HincII* enzyme. Expected fragment sizes were as follows: T/T: 300 bp; G/G: 200, 100; G/T: 300, 200, and 100 (Ming-Zhong et al., 2015). A – Representative cases. Lane M: 1000 kb DNA marker; Lane 1: G/G genotype; Lanes 3, 6, 7, and 9: G/T genotype; Lanes 2, 4, 5, and 8: T/T genotype. B – Representative controls. Lane M: 1000 kb DNA marker; Lane 4: G/T genotype; the rest are T/T genotype

**Table 2:** *XRCC4 c.1394G>T* Genotype and Allele Frequency Distribution among Breast Cancer Cases and Matched Clinically Healthy Controls

Genotype	Cases			Controls			p-values	OR* (95% CI)
	n	%	HWE*	n	%	HWE*		
G/G	1	0.97		0	0			1
G/T	34	33.01	0.147	16	15.53	0.392	**0.007**	**2.67 (1.36-5.25)**
T/T	68	66.02		87	84.87			0.357 (0.183-5.25)
G allele	36	17.48		16	7.77			
T allele	170	82.52		190	92.23		**0.003**	

*HWE, Hardy-Weinberg Equilibrium; *OR, Odds Ratio

**Table 3:** Combined Effect of *XRCC4* G/T Genotype with Lifestyle Factor and Family History of Cancer

Combination	Cases		Controls		p-value	OR (95% Cl)
	n	%	n	%		
*XRCC4* G/T and alcohol use	5	4.85	3	2.91	0.471	1.7 (0.3957 - 7.3102)
*XRCC4* G/T and tobacco use	2	1.94	1	0.971	0.561	1.98 (0.1803 - 22.6286)
*XRCC4* G/T and passive smoker	13	12.6	1	0.971	**0.001**	**14.73 (9.8898 - 18.8649)**
*XRCC4* G/T with history of cancer in immediate family	9	8.74	5	4.85	0.268	1.88 (0.6067 - 5.805)
*XRCC4* G/T with history of cancer in extended family	12	11.7	5	4.85	0.076	2.58 (0.8763 - 7.6231)

**Table 4:** Combined Effect of *XRCC4* T/T Genotype with Lifestyle Factor and Family History of Cancer

Combination	Cases		Controls		p-value	OR (95% Cl)
	n	%	n	%		
*XRCC4* T/T and alcohol use	11	10.7	24	23.3	0.016	0.39 (0.1814 - 0.8538)
*XRCC4* T/T and tobacco use	8	7.8	9	8.7	0.81	0.88 (0.3255 - 2.3769)
*XRCC4* T/T and passive smoker	43	41.7	14	13.6	**<0.001**	**4.55 (2.2938 - 9.0492)**
*XRCC4* T/T and history of cancer in immediate family	17	16.5	25	24.3	0.16	0.62 (0.3099 - 1.2273)
*XRCC4* T/T with history of cancer in extended family	24	23.3	26	25.2	0.74	0.9 (0.4756 - 1.0719)

Results show that genotypic (p=0.007) and allelic (p=0.003) distribution pattern of *XRCC4 c.1394G>T *were significantly different between breast cancer cases and healthy controls. It appears that G/T genotype (OR=2.67, 95% CI 1.36 – 5.25) is associated with breast cancer among Filipinos (Table 2). Moreover, cases who were exposed to passive smoking, whether carrying the *XRCC4 G/T* (OR=14.73, 95%CI 9.88-18.86) or T/T (OR=4.55, 95%CI 2.29-9.04) genotype, had increased risk for breast cancer (Table 3). Meanwhile, individuals carrying the T/T (OR=0.39, 95%CI 0.18-0.85) genotype and were also alcohol users seem to have lower risk of developing breast cancer compared to those carrying the G/T genotype (Table 4). 

## Discussion

Previous studies have associated the trinucleotide repeat polymorphism (CAGn) in the exon 1 of the androgen receptor gene (AR) (Liede et al., 2003) and mutations in *BRCA1* and *BRCA2* (De Leon Matsuda et al., 2002) with breast cancer among Filipino women. In contrast, variants of the* GSTM1* and *GSTT1* genes were not found to be risk factors for breast cancer development among the same population (Kalacas et al., 2019). Thus, identifying genes associated with cancer development continues to be a major goal of current research. 

*XRCC4*, which plays a role in both non-homologous end joining and completion of the V(D)J recombination, is among the genes that have been associated with various types of cancer (Krupa et al., 2011). The *XRCC4 c.1394G>T*, a mutation which lies at the coding region of the *XRCC4* gene, might cause an alteration in the amino acid sequence of the XRCC4 protein (Kabziński et al., 2015). This alteration might affect the function of XRCC4 protein in stabilizing the DNA ligase IV which is a crucial part in double strand break repair (Ming-zhong et al., 2015).

This study shows that heterozygous *G/T* genotype of *XRCC4 c.1394G>T* is associated with breast cancer in selected Filipino population. Similarly, *XRCC4 c.1394G>T G/T* genotype has been associated with breast, gastric, and prostate cancers among Taiwanese (Chiu et al., 2008) and colorectal cancer among Iranians (Emami et al., 2015). In contrast, the *XRCC4 c.1394G>T G/T* genotype was not associated with urinary bladder cancer among the Indians. Besides, the* G/G* genotype was associated with reduced risk for urothelial bladder in this population (Mittal, et al., 2012). In the current study, the *T/T* variant of *XRCC4 c.1394G>T* was associated with reduced risk for breast cancer. 

The present study also considered the combined effect of the genotype variants of *XRCC4 c.1394G>T *with clinical and epidemiologic risk factors. Among cases carrying the *G/T* genotype, those exposed to passive smoke had higher risk of breast cancer compared to active smokers. Inhaled side stream smoke, the main component of second-hand smoke, is about four times more toxic than active smoking as it contains about 69 known carcinogens and more particulate matter pollution that get into the air (Huang et al., 2005). 

To the researchers’ knowledge, this is the first report on the association of *XRCC4 c.1394G>T* with breast cancer development among selected Filipinos. Results of this study contribute to existing evidence supporting the hypothesis that polymorphisms in the *XRCC4 c.1394G>T *gene may influence the functioning of the DNA repair pathway. Despite the small number of participants in this study, the strict age- and sex-match design adds to the reliability of the findings. However, related future studies should screen for other polymorphisms in the *XRCC4 *gene and other DNA repair genes, and to determine their combined effects with lifestyle risk factors. Furthermore, participants from other parts of the country should be included to accurately represent the Filipino population. 

## Funding Statement

This study was supported by research grants from the Department of Science and Technology – Science Education Institute (DOST – SEI) and the Commission on Higher Education (CHED) of the Philippines.

## Statement conflict of Interest

The authors declare no conflict of interest
